# Supplementary material for: Handgrip and gait metrics as scalable markers of physical health in schizophrenia and alcohol use disorders
Source: Glob Ment Health (Camb). 2026 Apr 1;13:e86. doi: 10.1017/gmh.2026.10190 (PMC13150775; doi:10.1017/gmh.2026.10190)

**Supplementary Table 1. List of feature variables.**

| **Category** | **Features** | **Unit** | **Detail** |
| --- | --- | --- | --- |
| Handgrip | Absolute grip strength (HGS) | kg | predictor of muscular strength |
|  | Relative handgrip strength (rHGS) |  | absolute grip strength/BMI |
| WALK | Speed | m/s | time taken for a round trip of 12M at self-selected speed |
|  | Stride length | m | distance measured parallel to the line of progression, including two consecutive steps |
|  | Walk ratio | cm/steps/min | step length/cadence(number of steps/minute)* |
|  | Walk quality index | % | ratio of stance-swing phase in gait cycle** |
|  | Symmetry index | % | index for the left and right feet, a value closer to 100 indicates more symmetrical gait*** |
|  | Propulsion index | m/s² | force for forward propulsion during the single limb support phase of gait+ |
|  | Propulsion index difference |  | difference in left and right propulsive forces (absolute value)++ |
| TUG | Analysis duration | s | total time duration of whole TUG test |
|  | Phases duration-sit to stand | s | get up from a chair |
|  | Phases duration-forward gait | s | walk three meters |
|  | Phases duration-mid turning | s | turn around |
|  | Phases duration-return gate | s | walk back to the chair |
|  | Phases duration-end turning stand to sit | s | sit on the chair again |

* Considered an index of neuromotor control

** When it is 100%, the gait cycle on that side is evenly divided into 60% stance and 40% swing phases. In non-pathological subjects, the deviation is typically less than 10%.

*** An index evaluating left–right gait symmetry, representing the correspondence between cycles performed by the right and left lower limbs. The maximum value measured by the BTS G-Sensor is 100%, and an ideal score is above 90%, indicating no substantial asymmetry in propulsion.<Spatial-temporal parameters of gait: reference data and a statistical method for normality assessment> <NORMATIVE DATA OF KNEE JOINT MOTION AND GROUND REACTION FORCES IN ADULT LEVEL WALKING >

+ Affects gait speed<The independent effects of speed and propulsive force on joint power generation in walking> < Evaluation of measurements of propulsion used to reflect changes in walking speed in individuals poststroke> <Gait cycle: phases, muscles and joints involved. >

++ For this value, a score above 3 indicates mild asymmetry, and above 5 indicates severe asymmetry.

**Supplementary Table 2. Distribution of handgrip strength, WALK, and TUG across groups.**

|  |  | **HC^a^** | **AUD^b^** | **SCZ^c^** | **PT^b+c^** | **P-value** |
| --- | --- | --- | --- | --- | --- | --- |
|  |  | **(n = 210)** | **(n = 80)** | **(n = 144)** | **(n=224)** |  |
| Handgrip strength | HGS(kg) | 32.32 ± 10.15 | 30.35 ± 9.51 | 27.53 ± 10.97 | 28.54 ± 10.54 | 0.0002 |
|  | rHGS(HGS/BMI) | 1.38 ± 0.37 | 1.30 ± 0.41 | 1.08 ± 0.45 | 1.16 ± 0.44 | <0.0001 |
| WALK | Speed(m/s) | 1.20 ± 0.17 | 1.25 ± 0.21 | 1.19 ± 0.23 | 1.21 ± 0.22 | 0.5658 |
|  | Stride length(m) | 1.26 ± 0.17 | 1.29 ± 0.18 | 1.26 ± 0.20 | 1.27 ± 0.19 | 0.5466 |
|  | Walk Ratio(cm/steps/min) | 0.55 ± 0.09 | 0.56 ± 0.10 | 0.56 ± 0.11 | 0.56 ± 0.11 | 0.3204 |
|  | Walk quality index | 96.61 ± 2.34 | 95.71 ± 1.90 | 95.57 ± 2.54 | 95.62 ± 2.32 | <0.0001 |
|  | Symmetry index | 95.28 ± 4.19 | 92.69 ± 4.87 | 92.52 ± 6.04 | 92.58 ± 5.64 | <0.0001 |
|  | Propulsion index | 9.07 ± 1.95 | 8.58 ± 2.36 | 8.45 ± 2.37 | 8.49 ± 2.36 | 0.0057 |
|  | Propulsion index difference | 0.96 ± 0.77 | 1.23 ± 1.14 | 1.18 ± 1.00 | 1.20 ± 1.05 | 0.0069 |
| TUG | Analysis duration | 9.87 ± 1.36 | 10.44 ± 1.92 | 10.62 ± 2.27 | 10.55 ± 2.15 | 0.0001 |
|  | Phases duration-sit to stand(s) | 1.44 ± 0.24 | 1.44 ± 0.39 | 1.47 ± 0.30 | 1.46 ± 0.34 | 0.6040 |
|  | Phases duration-forward gait(s) | 2.30 ± 0.59 | 2.38 ± 0.66 | 2.49 ± 0.81 | 2.45 ± 0.76 | 0.0240 |
|  | Phases duration-mid turning(s) | 1.60 ± 0.42 | 1.83 ± 0.56 | 1.96 ± 0.66 | 1.92 ± 0.63 | <0.0001 |
|  | Phases duration-return gate(s) | 2.19 ± 0.56 | 2.28 ± 0.82 | 2.08 ± 0.90 | 2.15 ± 0.88 | 0.6224 |
|  | Phases duration-stand to sit(s) | 2.33 ± 0.54 | 2.51 ± 0.66 | 2.61 ± 0.74 | 2.58 ± 0.71 | 0.0001 |

**HC**, Healthy Control; **AUD**, Alcohol Use Disorder; **SCZ**, Schizophrenia; **PT**, Psychiatric Patients; **HGS**, hand grip strength; **rHGS**; relative hand grip strength

*P-*values were calculated using independent t-tests to estimate differences between the healthy control (HC) and psychiatric patient (PT) groups.

**Supplementary Table 3. Univariable and multivariable linear regression model**

|  | **Outcome** | **Comparison** | **Univariable model** | | | |
| --- | --- | --- | --- | --- | --- | --- |
|  |  |  | **Coefficient** | **95% CI** | **p (raw)** | **p (FDR)** |
| Handgrip strength | HGS(kg) | AUD - HC | -1.975 | (-4.639, 0.689) | 0.146 | 0.263 |
|  |  | SCZ - HC | -4.789 | (-6.982, -2.595) | <0.001 | <0.001 |
|  |  | SCZ - AUD | 2.814 | (-0.013, 5.641) | 0.051 | 0.115 |
|  | rHGS(HGS/BMI) | AUD - HC | -0.083 | (-0.188, 0.022) | 0.119 | 0.223 |
|  |  | SCZ - HC | -0.298 | (-0.384, -0.212) | <0.001 | <0.001 |
|  |  | SCZ - AUD | 0.215 | (0.104, 0.326) | <0.001 | 0.001 |
| WALK | Speed(m/s) | AUD - HC | 0.051 | (-0.001, 0.103) | 0.055 | 0.117 |
|  |  | SCZ - HC | -0.011 | (-0.054, 0.032) | 0.611 | 0.723 |
|  |  | SCZ - AUD | 0.062 | (0.007, 0.117) | 0.028 | 0.073 |
|  | Stride length(m) | AUD - HC | 0.030 | (-0.017, 0.077) | 0.214 | 0.357 |
|  |  | SCZ - HC | <0.001 | (-0.039, 0.039) | 0.993 | 0.993 |
|  |  | SCZ - AUD | 0.030 | (-0.02, 0.08) | 0.239 | 0.374 |
|  | Walk Ratio(cm/steps/min) | AUD - HC | 0.011 | (-0.015, 0.037) | 0.402 | 0.538 |
|  |  | SCZ - HC | 0.009 | (-0.013, 0.03) | 0.423 | 0.538 |
|  |  | SCZ - AUD | 0.002 | (-0.025, 0.03) | 0.866 | 0.890 |
|  | Walk quality index | AUD - HC | -0.894 | (-1.497, -0.291) | 0.004 | 0.015 |
|  |  | SCZ - HC | -1.034 | (-1.531, -0.537) | <0.001 | <0.001 |
|  |  | SCZ - AUD | 0.140 | (-0.5, 0.78) | 0.668 | 0.751 |
|  | Symmetry index | AUD - HC | -2.587 | (-3.878, -1.296) | <0.001 | 0.001 |
|  |  | SCZ - HC | -2.761 | (-3.824, -1.698) | <0.001 | <0.001 |
|  |  | SCZ - AUD | 0.174 | (-1.196, 1.544) | 0.803 | 0.861 |
|  | Propulsion index | AUD - HC | -0.489 | (-1.05, 0.072) | 0.088 | 0.171 |
|  |  | SCZ - HC | -0.624 | (-1.087, -0.162) | 0.008 | 0.031 |
|  |  | SCZ - AUD | 0.135 | (-0.461, 0.731) | 0.655 | 0.751 |
|  | Propulsion index difference | AUD - HC | 0.271 | (0.031, 0.51) | 0.027 | 0.073 |
|  |  | SCZ - HC | 0.222 | (0.025, 0.419) | 0.027 | 0.073 |
|  |  | SCZ - AUD | 0.049 | (-0.205, 0.303) | 0.705 | 0.774 |
| TUG | Analysis duration | AUD - HC | 0.571 | (0.103, 1.039) | 0.017 | 0.055 |
|  |  | SCZ - HC | 0.746 | (0.36, 1.132) | <0.001 | 0.001 |
|  |  | SCZ - AUD | -0.175 | (-0.672, 0.322) | 0.489 | 0.595 |
|  | Phases duration-sit to stand(s) | AUD - HC | -0.006 | (-0.082, 0.069) | 0.870 | 0.890 |
|  |  | SCZ - HC | 0.026 | (-0.036, 0.088) | 0.413 | 0.538 |
|  |  | SCZ - AUD | -0.032 | (-0.112, 0.048) | 0.430 | 0.538 |
|  | Phases duration-forward gait(s) | AUD - HC | 0.076 | (-0.1, 0.252) | 0.398 | 0.538 |
|  |  | SCZ - HC | 0.187 | (0.042, 0.332) | 0.012 | 0.040 |
|  |  | SCZ - AUD | -0.111 | (-0.298, 0.076) | 0.243 | 0.374 |
|  | Phases duration-mid turning(s) | AUD - HC | 0.226 | (0.087, 0.366) | 0.001 | 0.007 |
|  |  | SCZ - HC | 0.361 | (0.247, 0.476) | <0.001 | <0.001 |
|  |  | SCZ - AUD | -0.135 | (-0.282, 0.013) | 0.074 | 0.151 |
|  | Phases duration-return gate(s) | AUD - HC | 0.096 | (-0.095, 0.287) | 0.323 | 0.469 |
|  |  | SCZ - HC | -0.107 | (-0.265, 0.05) | 0.181 | 0.312 |
|  |  | SCZ - AUD | 0.204 | (0.001, 0.406) | 0.049 | 0.115 |
|  | Phases duration-stand to sit(s) | AUD - HC | 0.177 | (0.014, 0.341) | 0.034 | 0.084 |
|  |  | SCZ - HC | 0.279 | (0.145, 0.414) | <0.001 | <0.001 |
|  |  | SCZ - AUD | -0.102 | (-0.276, 0.072) | 0.249 | 0.374 |

**Supplementary Table 4. Univariable and multivariable linear regression models comparing handgrip strength, WALK, and TUG between healthy control (HC) and psychiatric patient (PT) groups.**

|  | **Outcome** | **Univariable model** | | | | **Multivariable model** | | | |
| --- | --- | --- | --- | --- | --- | --- | --- | --- | --- |
|  |  | **Coefficient** | **SE** | **95% CI** | **P-value** | **Coefficient** | **SE** | **95% CI** | **P-value** |
| Handgrip strength | HGS(kg) | -3.784 | 0.994 | (-5.738, -1.830) | <0.001 | -9.546 | 0.836 | (-11.189, -7.903) | <0.001 |
|  | rHGS(HGS/BMI) | -0.221 | 0.040 | (-0.299, -0.143) | <0.001 | -0.368 | 0.034 | (-0.436, -0.301) | <0.001 |
| WALK | Speed(m/s) | 0.011 | 0.019 | (-0.027, 0.049) | 0.569 | -0.021 | 0.022 | (-0.064, 0.022) | 0.332 |
|  | Stride length(m) | 0.011 | 0.018 | (-0.024, 0.045) | 0.548 | -0.038 | 0.018 | (-0.21, 0.021) | 0.035 |
|  | Walk Ratio(cm/steps/min) | 0.010 | 0.010 | (-0.009, 0.028) | 0.322 | -0.017 | 0.010 | (-0.098, 0.021) | 0.093 |
|  | Walk quality index | -0.984 | 0.224 | (-1.424, -0.543) | <0.001 | -0.885 | 0.266 | (-1.408, -0.363) | 0.001 |
|  | Symmetry index | -2.699 | 0.480 | (-3.642, -1.756) | <0.001 | -1.997 | 0.562 | (-3.91, -1.066) | <0.001 |
|  | Propulsion index | -0.576 | 0.209 | (-0.986, -0.166) | 0.006 | -0.008 | 0.241 | (-0.459, 1.173) | 0.973 |
|  | Propulsion index difference | 0.239 | 0.089 | (0.065, 0.414) | 0.007 | 0.205 | 0.105 | (-0.389, 2.251) | 0.053 |
| TUG | Analysis duration | 0.684 | 0.174 | (0.341, 1.026) | <0.001 | 0.476 | 0.202 | (0.04, 1.017) | 0.019 |
|  | Phases duration-sit to stand(s) | 0.014 | 0.028 | (-0.041, 0.07) | 0.608 | 0.012 | 0.033 | (-0.053, 0.078) | 0.712 |
|  | Phases duration-forward gait(s) | 0.147 | 0.066 | (0.018, 0.276) | 0.025 | 0.155 | 0.077 | (0.003, 0.306) | 0.045 |
|  | Phases duration-mid turning(s) | 0.313 | 0.052 | (0.211, 0.415) | <0.001 | 0.252 | 0.061 | (0.132, 0.373) | <0.001 |
|  | Phases duration-return gate(s) | -0.035 | 0.071 | (-0.175, 0.105) | 0.627 | -0.081 | 0.083 | (-0.245, 0.082) | 0.329 |
|  | Phases duration-stand to sit(s) | 0.243 | 0.061 | (0.123, 0.363) | <0.001 | 0.138 | 0.070 | (-0.143, 0.523) | 0.051 |

SE, standard error; CI, confidence interval; HGS, hand grip strength; rHGS; relative hand grip strength

**Supplementary Table 5. Cook’s distance–based sensitivity analysis of multivariable regression models for gait and relative handgrip strength features presented in Figure 4. Regression coefficients and p-values are shown before and after exclusion of influential observations to assess the robustness of group comparisons.**

|  | **Outcome** | **Comparison** | **N_raw** | **N_influential** | **Coef_before** | **Coef_after** | **p_before** | **p_after** | **Significance_changed** | **Sign_changed** |
| --- | --- | --- | --- | --- | --- | --- | --- | --- | --- | --- |
|  |  |  |  |  |  |  |  |  |  |  |
| Handgrip strength | rHGS(HGS/BMI) | AUD - HC | 434 | 19 | -0.083 | -0.086 | 0.119 | 0.095 | FALSE | FALSE |
|  |  | SCZ - HC | 434 | 19 | -0.298 | -0.324 | 0.000 | 0.000 | FALSE | FALSE |
|  |  | SCZ - AUD | 224 | 10 | -0.215 | -0.189 | 0.000 | 0.001 | FALSE | FALSE |
| WALK | Speed(m/s) | AUD - HC | 434 | 25 | 0.051 | 0.047 | 0.055 | 0.050 | FALSE | FALSE |
|  |  | SCZ - HC | 434 | 25 | -0.011 | -0.002 | 0.611 | 0.922 | FALSE | FALSE |
|  |  | SCZ - AUD | 224 | 11 | -0.062 | -0.047 | 0.048 | 0.098 | TRUE | FALSE |
|  | Stride length(m) | AUD - HC | 434 | 25 | 0.030 | 0.035 | 0.214 | 0.100 | FALSE | FALSE |
|  |  | SCZ - HC | 434 | 25 | 0.000 | 0.000 | 0.993 | 0.988 | FALSE | TRUE |
|  |  | SCZ - AUD | 224 | 10 | -0.030 | -0.031 | 0.268 | 0.213 | FALSE | FALSE |
|  | Walk Ratio(cm/steps/min) | AUD - HC | 434 | 16 | 0.011 | 0.013 | 0.402 | 0.270 | FALSE | FALSE |
|  |  | SCZ - HC | 434 | 16 | 0.009 | 0.013 | 0.423 | 0.180 | FALSE | FALSE |
|  |  | SCZ - AUD | 224 | 8 | -0.002 | -0.001 | 0.873 | 0.926 | FALSE | FALSE |
|  | Walk quality index | AUD - HC | 434 | 13 | -0.894 | -1.052 | 0.004 | 0.000 | FALSE | FALSE |
|  |  | SCZ - HC | 434 | 13 | -1.034 | -0.832 | 0.000 | 0.000 | FALSE | FALSE |
|  |  | SCZ - AUD | 224 | 7 | -0.140 | 0.058 | 0.667 | 0.827 | FALSE | TRUE |
|  | Symmetry index | AUD - HC | 434 | 27 | -2.587 | -1.606 | 0.000 | 0.000 | FALSE | FALSE |
|  |  | SCZ - HC | 434 | 27 | -2.761 | -1.847 | 0.000 | 0.000 | FALSE | FALSE |
|  |  | SCZ - AUD | 224 | 13 | -0.174 | 0.111 | 0.826 | 0.840 | FALSE | TRUE |
| TUG | Analysis duration | AUD - HC | 434 | 22 | 0.571 | 0.452 | 0.017 | 0.030 | FALSE | FALSE |
|  |  | SCZ - HC | 434 | 22 | 0.746 | 0.503 | 0.000 | 0.002 | FALSE | FALSE |
|  |  | SCZ - AUD | 224 | 7 | 0.175 | -0.007 | 0.560 | 0.976 | FALSE | TRUE |

The reference group in regression model is healthy control (HC); HGS, hand grip strength; rHGS; relative hand grip strength

**Supplementary Table 6. Sensitivity analysis of correlations between relative handgrip strength and gait features shown in Figure 4, comparing raw correlations and correlations after IQR-based outlier exclusion (1.5×IQR).**

|  | **Outcome** | **Comparison** | **r_raw** | **r_clean** | **n_raw** | **n_clean** | **n_outlier** | **abs_diff** |
| --- | --- | --- | --- | --- | --- | --- | --- | --- |
|  |  |  |  |  |  |  |  |  |
| WALK | Speed(m/s) | relative Grip | 0.297 | 0.325 | 434 | 429 | 5 | 0.028 |
|  | Stride length(m) | relative Grip | 0.430 | 0.429 | 434 | 427 | 7 | 0.001 |
|  | Walk Ratio(cm/steps/min) | relative Grip | 0.412 | 0.428 | 434 | 426 | 8 | 0.016 |
|  | Walk quality index | relative Grip | 0.055 | 0.085 | 434 | 422 | 12 | 0.031 |
|  | Symmetry index | relative Grip | 0.122 | 0.159 | 434 | 404 | 30 | 0.036 |
| TUG | Analysis duration | relative Grip | -0.265 | -0.284 | 434 | 427 | 7 | 0.019 |

**Supplementary Table 7. Sensitivity analysis of multivariable regression models using Cook’s distance to assess the influence of influential observations across handgrip strength, gait, and iTUG features. Regression coefficients and p-values are shown before and after exclusion of influential points for each group comparison.**

|  | **Outcome** | **Comparison** | **N_raw** | **N_influential** | **Coef_before** | **Coef_after** | **p_before** | **p_after** | **Significance_changed** | **Sign_changed** |
| --- | --- | --- | --- | --- | --- | --- | --- | --- | --- | --- |
|  |  |  |  |  |  |  |  |  |  |  |
| Handgrip strength | HGS(kg) | AUD - HC | 434 | 18 | -1.975 | -1.591 | 0.146 | 0.238 | FALSE | FALSE |
|  |  | SCZ - HC | 434 | 18 | -4.789 | -4.571 | 0.000 | 0.000 | FALSE | FALSE |
|  |  | SCZ - AUD | 224 | 5 | -2.814 | -2.957 | 0.055 | 0.041 | TRUE | FALSE |
|  | rHGS(HGS/BMI) | AUD - HC | 434 | 19 | -0.083 | -0.086 | 0.119 | 0.095 | FALSE | FALSE |
|  |  | SCZ - HC | 434 | 19 | -0.298 | -0.324 | 0.000 | 0.000 | FALSE | FALSE |
|  |  | SCZ - AUD | 224 | 10 | -0.215 | -0.189 | 0.000 | 0.001 | FALSE | FALSE |
| WALK | Speed(m/s) | AUD - HC | 434 | 25 | 0.051 | 0.047 | 0.055 | 0.050 | FALSE | FALSE |
|  |  | SCZ - HC | 434 | 25 | -0.011 | -0.002 | 0.611 | 0.922 | FALSE | FALSE |
|  |  | SCZ - AUD | 224 | 11 | -0.062 | -0.047 | 0.048 | 0.098 | TRUE | FALSE |
|  | Stride length(m) | AUD - HC | 434 | 25 | 0.030 | 0.035 | 0.214 | 0.100 | FALSE | FALSE |
|  |  | SCZ - HC | 434 | 25 | 0.000 | 0.000 | 0.993 | 0.988 | FALSE | TRUE |
|  |  | SCZ - AUD | 224 | 10 | -0.030 | -0.031 | 0.268 | 0.213 | FALSE | FALSE |
|  | Walk Ratio(cm/steps/min) | AUD - HC | 434 | 16 | 0.011 | 0.013 | 0.402 | 0.270 | FALSE | FALSE |
|  |  | SCZ - HC | 434 | 16 | 0.009 | 0.013 | 0.423 | 0.180 | FALSE | FALSE |
|  |  | SCZ - AUD | 224 | 8 | -0.002 | -0.001 | 0.873 | 0.926 | FALSE | FALSE |
|  | Walk quality index | AUD - HC | 434 | 13 | -0.894 | -1.052 | 0.004 | 0.000 | FALSE | FALSE |
|  |  | SCZ - HC | 434 | 13 | -1.034 | -0.832 | 0.000 | 0.000 | FALSE | FALSE |
|  |  | SCZ - AUD | 224 | 7 | -0.140 | 0.058 | 0.667 | 0.827 | FALSE | TRUE |
|  | Symmetry index | AUD - HC | 434 | 27 | -2.587 | -1.606 | 0.000 | 0.000 | FALSE | FALSE |
|  |  | SCZ - HC | 434 | 27 | -2.761 | -1.847 | 0.000 | 0.000 | FALSE | FALSE |
|  |  | SCZ - AUD | 224 | 13 | -0.174 | 0.111 | 0.826 | 0.840 | FALSE | TRUE |
|  | Propulsion index | AUD - HC | 434 | 21 | -0.489 | -0.845 | 0.088 | 0.001 | TRUE | FALSE |
|  |  | SCZ - HC | 434 | 21 | -0.624 | -0.681 | 0.008 | 0.001 | FALSE | FALSE |
|  |  | SCZ - AUD | 224 | 9 | -0.135 | 0.046 | 0.682 | 0.875 | FALSE | TRUE |
|  | Propulsion index difference | AUD - HC | 434 | 23 | 0.271 | 0.015 | 0.027 | 0.880 | TRUE | FALSE |
|  |  | SCZ - HC | 434 | 23 | 0.222 | 0.075 | 0.027 | 0.340 | TRUE | FALSE |
|  |  | SCZ - AUD | 224 | 11 | -0.049 | 0.037 | 0.740 | 0.756 | FALSE | TRUE |
| TUG | Analysis duration | AUD - HC | 434 | 22 | 0.571 | 0.452 | 0.017 | 0.030 | FALSE | FALSE |
|  |  | SCZ - HC | 434 | 22 | 0.746 | 0.503 | 0.000 | 0.002 | FALSE | FALSE |
|  |  | SCZ - AUD | 224 | 7 | 0.175 | -0.007 | 0.560 | 0.976 | FALSE | TRUE |
|  | Phases duration-sit to stand(s) | AUD - HC | 434 | 16 | -0.006 | -0.055 | 0.870 | 0.075 | FALSE | FALSE |
|  |  | SCZ - HC | 434 | 16 | 0.026 | -0.015 | 0.413 | 0.556 | FALSE | TRUE |
|  |  | SCZ - AUD | 224 | 5 | 0.032 | 0.078 | 0.495 | 0.033 | TRUE | FALSE |
|  | Phases duration-forward gait(s) | AUD - HC | 434 | 20 | 0.076 | 0.027 | 0.398 | 0.714 | FALSE | FALSE |
|  |  | SCZ - HC | 434 | 20 | 0.187 | 0.078 | 0.012 | 0.185 | TRUE | FALSE |
|  |  | SCZ - AUD | 224 | 11 | 0.111 | 0.058 | 0.295 | 0.487 | FALSE | FALSE |
|  | Phases duration-mid turning(s) | AUD - HC | 434 | 13 | 0.226 | 0.183 | 0.001 | 0.001 | FALSE | FALSE |
|  |  | SCZ - HC | 434 | 13 | 0.361 | 0.305 | 0.000 | 0.000 | FALSE | FALSE |
|  |  | SCZ - AUD | 224 | 7 | 0.135 | 0.114 | 0.126 | 0.086 | FALSE | FALSE |
|  | Phases duration-return gate(s) | AUD - HC | 434 | 18 | 0.096 | 0.000 | 0.323 | 0.999 | FALSE | FALSE |
|  |  | SCZ - HC | 434 | 18 | -0.107 | -0.170 | 0.181 | 0.008 | TRUE | FALSE |
|  |  | SCZ - AUD | 224 | 12 | -0.204 | -0.210 | 0.096 | 0.024 | TRUE | FALSE |
|  | Phases duration-stand to sit(s) | AUD - HC | 434 | 25 | 0.177 | 0.158 | 0.034 | 0.037 | FALSE | FALSE |
|  |  | SCZ - HC | 434 | 25 | 0.279 | 0.230 | 0.000 | 0.000 | FALSE | FALSE |
|  |  | SCZ - AUD | 224 | 10 | 0.102 | 0.045 | 0.305 | 0.606 | FALSE | FALSE |

The reference group in regression model is healthy control (HC); HGS, hand grip strength; rHGS; relative hand grip strength

**Supplementary Figure 1. Scatter plot showing the association between relative handgrip strength (rHGS) and gait features in the healthy control (HC) group.**


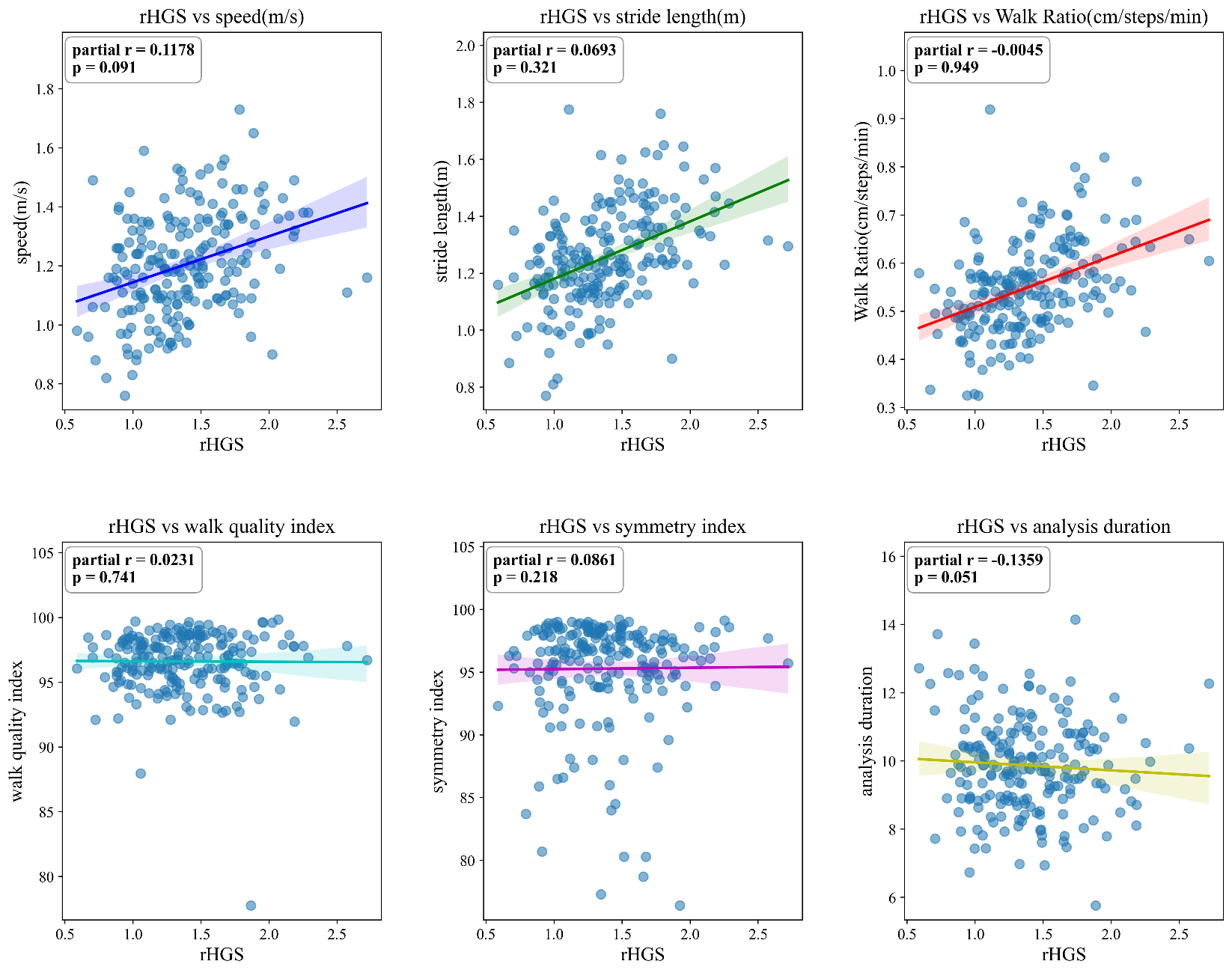


**Supplementary Figure 2. Scatter plot showing the association between relative handgrip strength (rHGS) and gait features in the schizophrenia (SCZ) group.**


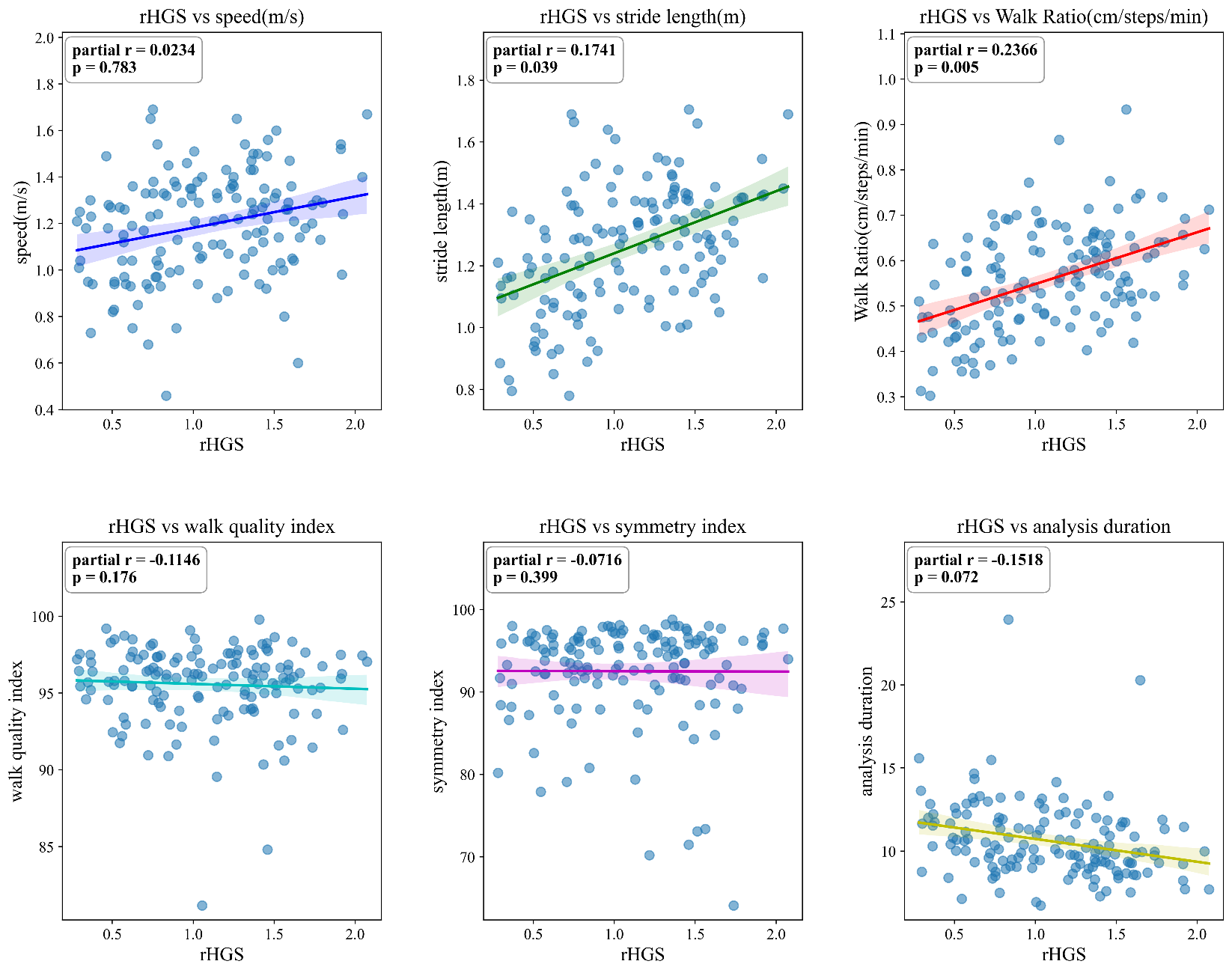


**Supplementary Figure 3. Scatter plots showing the relationships between relative handgrip strength (rHGS) and gait-related parameters across all participants. Data points are color-coded by diagnostic group (HC, AUD, SCZ). Group-specific linear regression lines are overlaid, and Pearson’s correlation coefficients with corresponding p-values are shown for each group. This visualization complements the group-wise analyses presented in Figure 4 by illustrating between-group heterogeneity in association patterns.**


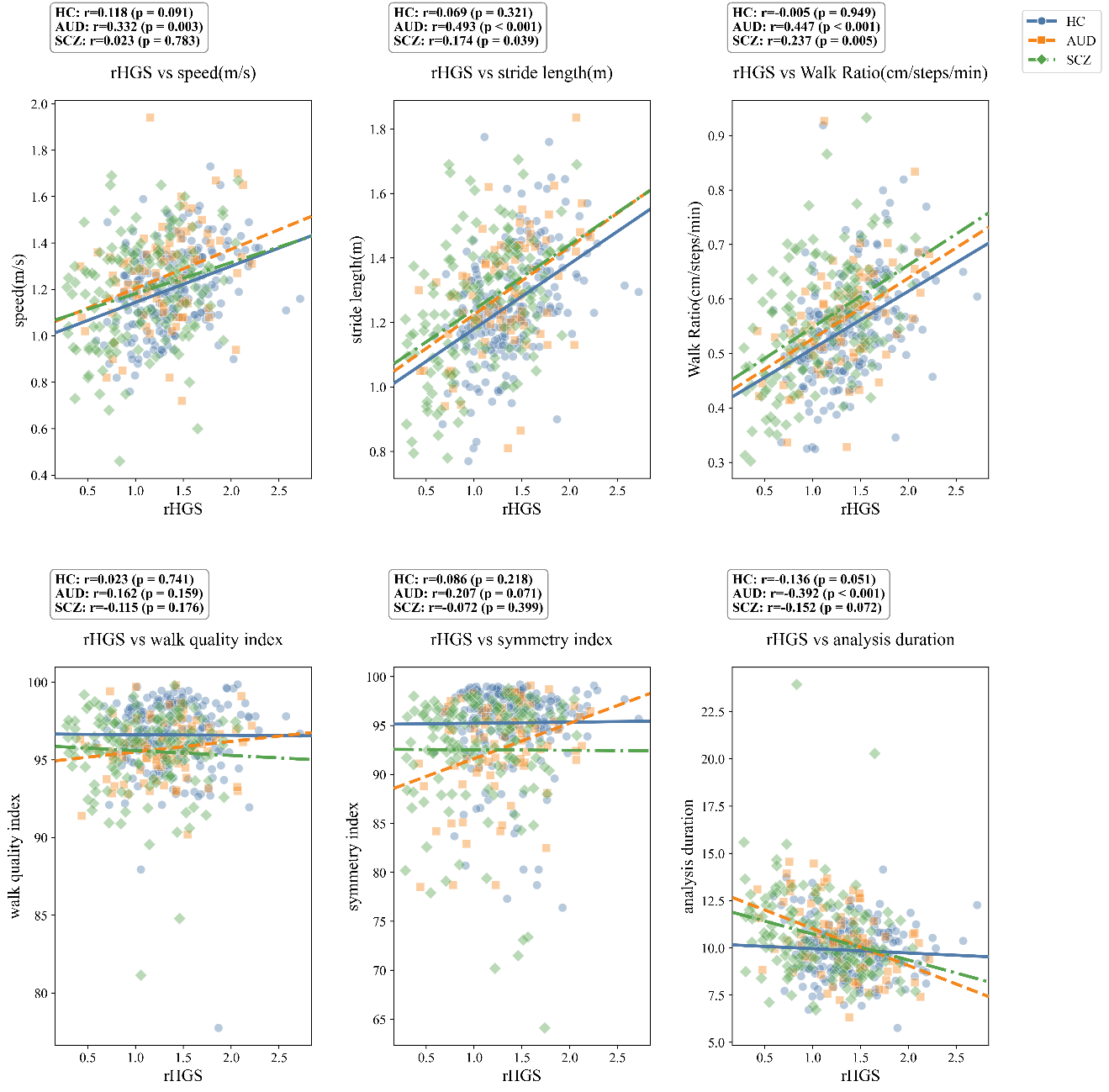

Supplement: Park et al. supplementary material [file S2054425126101903sup001.docx]
